# Sex-Specific Responses of Sexual Reproduction, Clonal Reproduction, and Vegetative Growth to Environmental (Biotic and Abiotic) Factors in the Clonal Dioecious Plant *Acer barbinerve*

**DOI:** 10.3390/plants14040596

**Published:** 2025-02-15

**Authors:** Dan Liao, Jingjing Lei, Yingni Wang, Yuxin Bao, Xinna Zhang, Juan Wang

**Affiliations:** 1 Key Laboratory for Forest Resources & Ecosystem Processes of Beijing, School of Ecology and Nature Conservation, Beijing Forestry University, Beijing 100083, China; liaodan@bjfu.edu.cn (D.L.); 18294324528@163.com (J.L.); wangyingni08042@163.com (Y.W.); baoyx0283@163.com (Y.B.); 2 College of Forestry, Beijing Forestry University, Beijing 100083, China; zhangxinna@bjfu.edu.cn

**Keywords:** environmental adaptation, sexual dimorphism, clonal plant, dioecy

## Abstract

Sexual dimorphism in dioecious plants serves as a critical adaptive strategy in complex environments. This study systematically investigated the effects of topographic factors (elevation, slope, aspect, and convexity), soil nutrients (C, N, P), and interspecific competition intensity on the reproductive strategies and vegetative growth of the clonal dioecious plant, *Acer barbinerve*. Using Spearman’s correlation analysis, multiple regression models, and PLS-PM path models, key findings include the following: (1) female sexual reproduction biomass showed a significant positive correlation with the topography principal component (topo_PC1), with a notable gender–topography interaction, whereas male sexual reproduction was negatively regulated by elevation; (2) clonal reproduction in both sexes was significantly suppressed by interspecific competition, but females additionally exhibited positive topographic responses; and (3) male vegetative growth was significantly impacted by environmental stress, while females maintained relative stability. These results demonstrate that females optimize reproductive investment through topography-mediated resource acquisition, whereas males are more susceptible to resource competition constraints. This sex-specific adaptive strategy corroborates the dimorphic niche hypothesis, highlighting how environmental heterogeneity drives divergent life history allocations in dioecious species. The findings provide novel insights into the ecological mechanisms underlying sexual dimorphism and inform gender ratio management in ecological restoration practices.

## 1. Introduction

Dioecious plants play an important role in terrestrial ecosystems [1]. Their sexual dimorphism reflects adaptive traits associated with reproductive roles [2,3]. The ecological, morphological, and physiological differences between sexes in dioecious plants are often attributed to varying reproductive costs of each sex and are closely related to the trade-offs assigned to reproductive and other plant functions [4,5]. Throughout their life cycle, plants are continuously influenced by both biotic and abiotic factors [6], and different sexes often respond differently to environmental changes. For example, females of *Populus euphratica* are more sensitive to drought and salinity than males [7], while male *Salix matsudana* exhibit stronger salt tolerance [8]. Therefore, understanding how environmental factors (both biotic and abiotic) affect the plant growth and reproduction of dioecious plants is crucial [9,10,11]. However, studies on the impact of environmental factors on dioecious plants still have two main limitations: a lack of systematic comparisons and limited consideration of multifactorial interactions [12,13].

According to the dimorphic niche hypothesis, differences in reproductive costs and allocation trade-offs between females and males may lead to their different responses to environmental gradients [14], and abiotic factors related to geography also play a role in modifying sexual dimorphism in plant life history [15]. Topographic features such as slope, aspect, and elevation are considered the primary factors causing spatial variations [16], and they promote the formation of environmental heterogeneity by regulating conditions such as soil moisture, light, and microclimate [17,18,19]. This heterogeneity further leads to differences in vegetative growth and reproduction processes [16,20,21]. In addition, plant growth and reproduction are limited by the availability of key elements (e.g., carbon, nitrogen, and phosphorus) in the environment [22,23]. For dioecious plants, there are differences in resource requirements between males and females [24]. For example, male plants may require more nitrogen for pollen production during flowering, whereas female plants require more carbon for seed production during fruiting [25]. Thus, sexual dimorphism exists in dioecy in response to topography and soil nutrients during growth and reproduction.

De Lisle demonstrated that resource competition drives the evolution of sexual dimorphism [26], which implies that there is a gender difference in the response of the two sexes to interspecific competition [27,28]. Plants compete for resources needed for growth and reproduction through interspecific competition in environments with limited resources. This competition usually drives plants to make morphological and physiological adaptations [29,30]. Different plant species respond differently to resource competition, and clonal plants in particular are more complex regarding interspecific competition due to their dual ability for both clonal growth and sexual reproduction. The resource allocation of clonal dioecious plants is often strongly influenced by interspecific competition pressures [31,32]. Therefore, studying the adaptation of dioecious plants under different interspecific competition intensities, especially the manifestation of sexual reproduction, clonal reproduction, and vegetative growth, is important for understanding plant growth and reproductive strategies in complex environments.

This study focuses on *Acer barbinerve* and systematically investigates the combined effects of abiotic factors (such as topography and soil nutrients) and biotic factors (such as interspecific competition) on its sexual reproduction, clonal reproduction, and vegetative growth. *Acer barbinerve* is a typical clonal dioecious plant in the Aceraceae family and is one of the foundational species in the temperate forests of China. It has high species diversity in its local area and is also one of the dominant understory species [33,34]. The sex of this plant can be easily distinguished during the flowering period (April to May), with the fruiting period occurring in July to August. As a root-suckering clonal plant, *Acer barbinerve* expands horizontally through underground rhizomes and forms new buds at certain nodes, which eventually develop into independent individuals. Due to the small spacing between its ramets, they can grow closely together, making it easier to identify the genets in the field. This study will address the following scientific questions: (1) Do abiotic factors have sexually dimorphic effects on *Acer barbinerve*? (2) Do biotic factors have sexually dimorphic effects on *Acer barbinerve*? (3) Are the differences in sexual dimorphism consistent across sexual reproduction, clonal reproduction, and vegetative growth?

## 2. Results

### 2.1. The Effects of Biotic and Abiotic Factors on Sexual Reproduction

**Figure 1:**
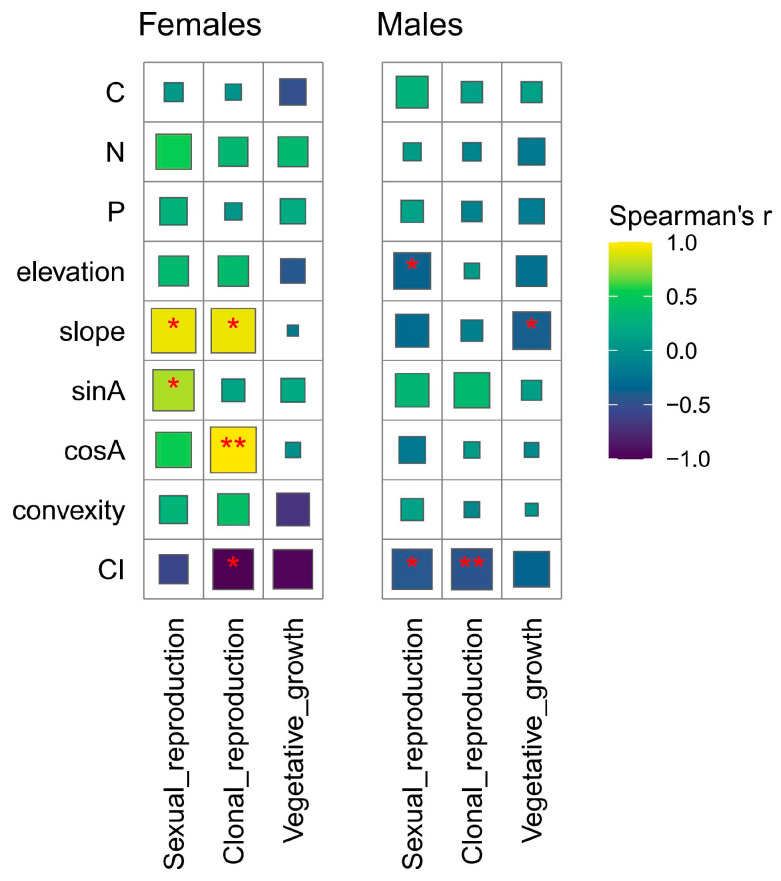
Correlations between sexual reproduction, clonal reproduction, vegetative growth, and their explanatory variables. The size of the squares indicates the significance of Spearman’s correlation between explanatory variables, and the colors represent the different strengths of the correlations. C, soil carbon content; N, soil nitrogen content; P, soil phosphorus content; sinA, east aspect; cosA, north aspect; CI, interspecific competition intensity. * *p* < 0.05; ** *p* < 0.01.

**Figure 2:**
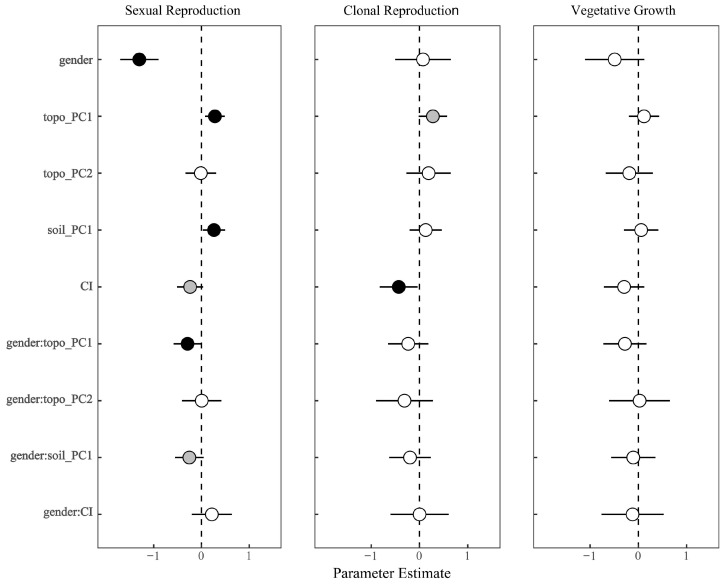
Effects of environmental variables on sexual reproduction, clonal reproduction, and vegetative growth. We show the parameter estimates of model predictors and the associated 95% confidence intervals. The *p*-values of each predictor are given as: Black indicates *p* < 0.05, grey indicates 0.05 *p* < 0.1, and white indicates *p* > 0.1. Gender, based on males; topo_PC1, the first axis of the five topography variables; topo_PC2, the second axis of the five topography variables; soil_PC1, the first axis of the four soil nutrients variables; CI, interspecific competition; gender:topo_PC1, the interaction between gender and the first axis of the five topography variables; gender:topo_PC2, the interaction between gender and the second axis of the five topography variables; gender:soil_PC1, the interaction between gender and the first axis of the four soil nutrients variables; gender:CI, the interaction between gender and interspecific competition.

**Figure 3:**
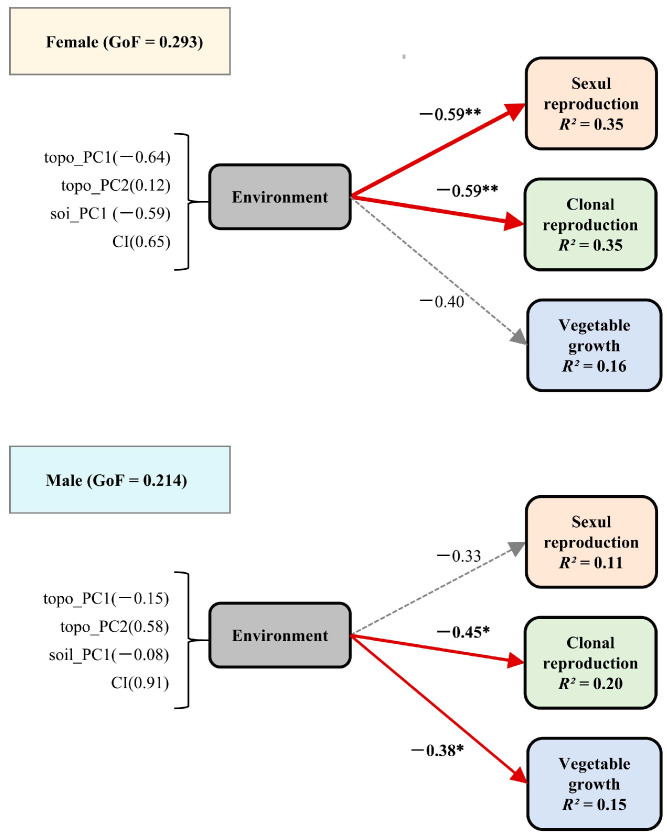
Structural equation model about the impact of the environment on sexual reproduction, clonal reproduction, and vegetative growth. Each number in parentheses indicates the loading value of the indicator to the latent variable. Red lines indicate negative significant relationships, respectively, and gray lines indicate insignificant relationships; the thickness of the line represents the strength of the causal relationship, supplemented by a standardized path coefficient. *R^2^* indicates the total variation of a dependent variable as explained by independent variables; GOF indicates the goodness of fit of the entire model. * *p* < 0.05; ** *p* < 0.01.

In the Spearman’s correlation analysis, slope and sinA are significantly positively correlated with sexual reproduction in females, while sexual reproduction in males is significantly negatively correlated with elevation and interspecific competition intensity (Figure 1). Other environmental factors, although showing some correlation, do not exhibit significant relationships. The multiple regression model for sexual reproduction (Figure 2) indicates that gender (β = −1.30, *p* < 0.001), topo_PC1 (β = 0.28, *p* < 0.01), and soil_PC1 (β = 0.26, *p* < 0.05) have significant effects on sexual reproduction biomass. Additionally, a significant interaction effect between gender and topo_PC1 was observed (β = −0.29, *p* < 0.05), suggesting that the influence of topo_PC1 on sexual biomass is weaker in males compared to females. A marginal interaction effect between gender and soil_PC1 was also found (*p* < 0.1). In the PLS-PM models (Figure 3), the environmental factor exhibits a significant negative effect on female sexual reproduction (β = −0.60, *p* < 0.01), while its negative impact on male sexual reproduction is not statistically significant (β = −0.33, *p* > 0.05).

### 2.2. The Effects of Biotic and Abiotic Factors on Clonal Reproduction

In the Spearman’s correlation analysis, clonal reproduction in females is significantly positively correlated with slope and cosA, and significantly negatively correlated with interspecific competition intensity. However, clonal reproduction in males is only significantly negatively correlated with interspecific competition intensity (Figure 1). The multiple regression model for clonal reproduction (Figure 2) reveals that gender does not have a significant effect on rematnumber (*p* = 0.80). However, CI exhibits a significant negative effect on rematnumber (β = −0.43, *p* < 0.05). Moreover, no significant interaction effects between the variables and gender were observed for number of ramets. In the PLS-PM models (Figure 3), the environment has a significant negative effect on female clonal reproduction (β = −0.60, *p* < 0.01), and it also exerts a significant negative influence on male sexual reproduction (β = −0.45, *p* < 0.05).

### 2.3. The Effects of Biotic and Abiotic Factors on Vegetative Growth

In the Spearman’s correlation analysis, vegetative growth in females does not show significant correlations with environmental factors, while vegetative growth in males is significantly negatively correlated only with slope (Figure 1). The multiple regression model for vegetative growth (Figure 2) indicates that gender does not have a significant effect on plant biomass (*p* = 0.12). The independent variables, including CI, topo_PC1, topo_PC2, and soil_PC1, also do not exhibit significant effects on plant biomass. Furthermore, no significant interaction effects, such as those between gender and the independent variables, were observed. In the PLS-PM models (Figure 3), the path coefficient for the environmental factor on female vegetative growth is −0.400 with a *p*-value of 0.08, which is close to the significance threshold of 0.05, suggesting a potential negative impact of the environment on vegetative growth, though this effect is not fully significant. In contrast, the environment exerts a significant negative effect on male vegetative growth (β = −0.38, *p* < 0.05).

## 3. Discussion

**Figure 4:**
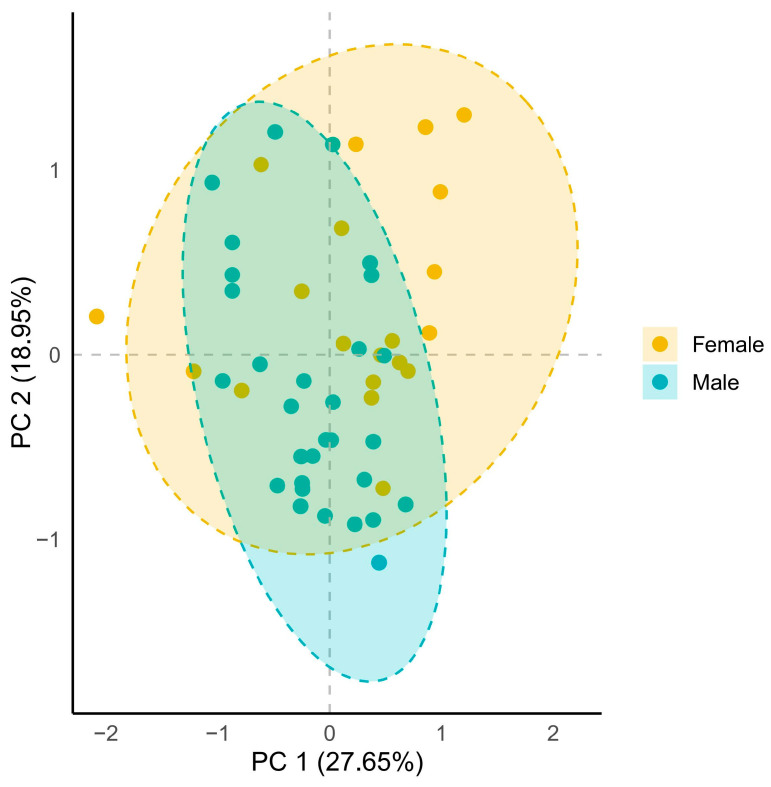
Principal component analysis of the topography, soil nutrients, and interspecific competition intensity of *Acer barbinerve* genets.

Female and male plants experience different selective pressures and have different evolutionary directions. Their evolutionary differences can induce a range of morphological, physiological, and ecological differences between males and females, which are referred to as sexual dimorphism. Sexual dimorphism is not fixed under different environmental pressures, and males and females differ in their plastic responses to environmental factors [35]. In our study, although principal component analyses showed no significant differences between female and male *Acer barbinerve* on environmental factors such as topography, soil nutrients, and interspecific competition intensity, this result suggests that overall variation in these environmental factors does not directly distinguish between the sexes in terms of growth and reproduction on a macro scale (Figure 4). However, subsequent analyses revealed significant sexual differences in the response to these environmental factors. This indicates that, despite the overall similarity of environmental factors, male and female *Acer barbinerve* exhibit sex-specific responses to growth and reproduction.

### 3.1. Differences in the Response of Sexual Reproduction to Environmental Factors Between the Sexes

This study reveals that sexual reproduction in females and males responds differently to environmental factors. Topography and soil nutrients have a significant impact on sexual reproduction, with a notable interaction between topography and gender. Topographic factors are critical in creating habitat differences across regions [36]. At small scales, elevation and slope are two of the most important topographic factors influencing plant functional traits [23]. In the response of three grassland plants to changes in elevation, it was found that the reproduction of *Trifolium montanum* and *Briza media* was almost unaffected by elevation, whereas *Ranunculus bulbosus* showed a significant increase in reproductive investment at higher elevations [37]. Olejniczak et al. also demonstrated a species-specific dependence of seed production on changes in elevation [38]. In this study, the interaction between sex and topo_PC1 suggests a weak effect of topographic factors on males This may be related to differences in water demand or water use efficiency between the sexes [39,40,41]. In addition to elevation and slope, aspect also affects sexual reproduction in plants. For the dioecious shrub *Bursera fagaroides* (Burseraceae), reproduction was found to be related to aspect, with individuals growing on west-facing slopes producing lower fruit production than those growing on east-facing slopes [42,43].

Male and female plants face different resource limitations during sexual reproduction, with female functions (i.e., seed and fruit production) generally requiring more carbon [25,44], while male functions may have higher nitrogen demands for pollen production [45,46], which in turn leads to differences in their nutrient requirements and resource allocation. Different resource currencies (e.g., carbon vs. nitrogen) in dioecious plants may be an important factor driving sex-specific patterns of sexual dimorphism [47]. Sexual reproduction in males is limited to flowering and pollen dispersal, while females continue with fertilization, seed, and fruit production and dispersal [48]. This study found that the sexual reproduction of males was significantly enhanced with increasing soil nutrients. The increase in soil nutrient content enhanced nitrogen uptake by male flowers, directing more nitrogen toward pollen production, which subsequently increased male flower production. This, in turn, improved the fertilization success rate of female flowers, ultimately leading to a higher number of successfully developed samaras.

In a study of the dioecious wind-pollinated shrub *Leucadendron rubrum*, it was found that plant density negatively impacted the male fitness proxy in males but not significantly in females [49]. Similarly, our study found that sexual reproduction in males was significantly suppressed by the intensity of interspecific competition, whereas females were unaffected. The negative effect of interspecific competition on male sexual reproduction may be due to the increased competition for nutrient resources that affect pollen production. Females showed a stronger sexual reproduction in high competitive environments, possibly because female plants prioritize investing more resources in seed development to ensure the reproductive success of their offspring [27,50].

These results suggest that although male and female *Acer barbinerve* grow in similar environments, their sexual reproduction varies in response to environmental factors. These sex-specific response patterns reflect differences in reproductive strategies and reveal the ecological adaptation strategies of different sexes in varying environments.

### 3.2. Differences in the Response of Clonal Reproduction to Environmental Factors Between the Sexes

Plants have repeatedly evolved clonal reproduction alongside sexual reproduction [51], forming an integrated strategy designed to ensure the transmission of adaptive genes while also providing the genetic variability necessary for colonizing new habitats and surviving future environmental changes [52]. In nature, the availability of basic resources (e.g., light, water, and soil nutrients) typically exhibits spatial heterogeneity.

Clonal plant ramets are often physically connected for extended periods through horizontal connectors (stolons and rhizomes), allowing for physiological integration between ramets [53,54]. Due to clonal integration, resources obtained by ramets growing in favorable microhabitats can be transported to resource-scarce locations, alleviating resource shortages in impoverished microsites [55]. Clonal plants can exhibit foraging responses, in which more resource-absorbing organs (e.g., leaves, roots, or ramets) are placed in high-quality patches than in low-quality ones, enabling the efficient use of heterogeneously distributed light and water resources [56,57]. Such cooperative systems can buffer the effects of spatial heterogeneity [58], and improve the overall performance of the plant [32,59].

It is due to these characteristics of clonal plants that topography and soil nutrient content are not the major factors influencing the clonal reproduction of *Acer barbinerve* under natural field conditions. Similarly to our study, when examining the reproductive allocation of dioecious aquatic clonal plants in response to water depth, it was found that both sexes invested similarly in clonal reproduction (via tubers) [47]. In general, the importance of clonal reproduction tends to increase with elevation. At higher elevations (3944 m), *Fragaria vesca* showed stronger clonal integration effects in terms of biomass, total stolon length, and number of ramets [60]. In a study on the variation of sexual and clonal reproduction in the alpine pioneer plant *Geum reptans* at different elevations, it was found that clonal reproduction was more common in low- and high-elevation populations, but less frequent in mid-elevation populations [61]. At the same time, the proportion of sexual and clonal reproduction in *Geum reptans* was more determined by the plastic responses of individual plants to local environmental conditions, rather than being solely driven by environmental gradients (e.g., elevation, etc.) [61]. This further emphasizes the importance of integrative studies.

In addition to resource heterogeneity, clonal plants are affected by many other environmental stressors, of which interspecific competition is an important factor. Studies have shown that survival, clonal reproduction, and most growth traits of clonal plants are reduced by competition [62]. Furthermore, the reproductive output of all plants (whether clonal or not) increases with improved habitat nutrient status and light conditions, while it declines under unfavorable moisture conditions [63]. The proportion of clonal plants usually varies across habitat gradients, which may be due to the fact that sexual reproduction becomes more difficult under certain environmental conditions, and clonal plants are able to bypass the barriers to sexual reproduction through clonal reproduction, thus gaining an adaptive advantage [64]. In clonal dioecious plants, resource allocation patterns may differ between the sexes: males typically allocate more resources to clonal structures, while females allocate less [3,65]. However, in the absence of competition, male and female plants of *Antennaria dioica* do not show significant differences in resource allocation [66]. In this study, clonal reproduction in both female and male *Acer barbinerve* was significantly negatively impacted by the intensity of interspecific competition, possibly because there was no significant difference in resource allocation for clonal reproduction between the sexes. Similarly, in the presence of *Phragmites australis*, the ramet density and rhizome sprouting rate of *Typha* spp. significantly decreased, indicating that interspecific competition negatively affected the clonal reproduction of *Typha* spp. [31].

### 3.3. Differences in the Response of Vegetative Growth to Environmental Factors Between the Sexes

Both biotic and abiotic factors affect biomass accumulation [67]. Competition alters the availability of nutrients, water, and light, thereby affecting plant growth and community structure in a given environment [9]. In this study, the vegetative growth of males was significantly negatively affected by environmental factors. Similarly to our study, Eppley found that females were more competitive than males in growth at high densities [68]. The presence of competition reduces plant biomass [69]. In studies on the impact of *Phragmites australis* invasion on *Typha* spp., it was found that the underground biomass of both plants decreased [31]. Consistent with previous studies, in this research, as the intensity of interspecific competition increased, the nutrient biomass of both sexes showed a declining trend.

Plants exhibit local adaptation through adaptive plasticity, altering their morphological or physiological traits along environmental gradients [70]. A recent study has shown that plant biomass follows a unimodal relationship with elevation, with resources concentrated in mid-elevation areas [71,72]. No significant relationship was found between the nutrient biomass of female and male plants with elevation in this study, probably because of the narrow range of elevation (435.47–501.96 m), which was not enough to show a significant elevation gradient effect, or because the environmental changes in this range had a more limited effect on plant growth and failed to cause a significant difference in biomass. Slope and aspect affect the amount of solar radiation received by vegetation surfaces, and solar radiation is a major component of surface energy balance, directly influencing microclimate conditions (e.g., near-surface temperature, evaporative demand, soil moisture, etc.) [20]. In mid-latitude regions, aspect has the most significant effect on ecological processes and soil properties [16,19]. North-facing slopes (shady slopes) typically have thicker, denser vegetation and nutrient-rich soils, while south-facing slopes (sunny slopes) have sparser vegetation, poorer soil development, and higher erosion rates. On south-facing slopes, species with a higher leaf mass area ratio (LMA) and smaller leaves are dominant, while on north-facing slopes, species with a lower LMA and larger leaves dominate [73]. Topography has a significant effect on tree growth, with trees growing faster in valleys than on ridges [74]. In this study, soil nutrient content had a relatively low impact on the vegetative growth of both female and male *Acer barbinerve*, indicating that the soil nutrient content in this area is generally sufficient to meet the vegetative growth needs of *Acer barbinerve*, and is not a limiting factor for vegetative growth.

## 4. Materials and Methods

### 4.1. Overview of the Study Area

This study was carried out in a natural temperate forest located in the Jiaohe Management Bureau of the Experimental Forest Zone in Jilin Province, northeastern China (43.97° N, 127.71–127.72° E). The area is characterized by a temperate continental climate, influenced by the monsoon, with an average annual temperature of 3.8 °C and an annual precipitation of 695.9 mm. The average monthly temperature ranges from −18.6 °C (January) to 21.7 °C (July). The soil type is mountainous dark brown forest soil with a thickness of 20–100 cm. In July 2009, a permanent forest monitoring study was initiated in the area, covering an area of 11.52 hm^2^ (320 m × 360 m) [75]. The study area is subdivided into 272 contiguous plots, each measuring 20 m × 20 m. All woody plant individuals with diameters at breast height (DBH) ≥ 1 cm were identified, labeled, measured, and located at the species level. The first survey of the study area was completed in 2009, and re-measurements were conducted every five years using the same methodology. The forest stand is classified as near-mature, with the dominant tree species in the main canopy including *Fraxinus mandshurica*, *Tilia amurensis*, *Pinus koraiensis*, *Quercus mongolica*, *Acer pictum* subsp. *mono*, *Ulmus davidiana* var. *japonica*, *Carpinus cordata*, and *Juglans mandshurica*. The understory is mainly composed of *Syringa reticulata* subsp. *amurensis*, *Corylus mandshurica*, and *Acer barbinerve* [33].

### 4.2. Survey Sampling

During the flowering period (May 2023), female and male *Acer barbinerve* genets were randomly selected in the study area. Each genet consisted of several ramets, and the basal survey was carried out on each ramet of the genet. During the flowering period, the numbers of female and male flowers were counted, and the corresponding floral biomass was calculated. During the fruiting period (July 2023), a follow-up basic survey was conducted on the selected genets. The number of ramets, number of leaves, length of new branches, and number of samaras (only for female plants) were recorded, and the corresponding biomass was calculated. Genets that died during the survey period were excluded. The final number of surviving genets at the fruiting period was 20 female plants and 30 male plants. The basic distribution of the genets is shown in Table S1.

For each ramet, 5–10 representative branches were marked at different orientations to reflect the average condition of the ramet. During the flowering and fruiting periods, the number of flowers, fruits, and leaves, and the length of new branches on the marked branches and the total number of branches of the ramets were counted. From these, the total numbers of flowers, fruits, and leaves, and the total new branch length for the plant were calculated. Corresponding samples of flowers, fruits, leaves, and new branches were collected. The flower, fruit, and leaf samples were dried to a constant weight at 65 °C, and then weighed using an analytical balance (AUW220D, Shimadzu, Shanghai, China) to determine their dry biomass. This allowed for the calculation of the biomass per flower, fruit, and leaf, and the biomass of new branches per unit length. By multiplying these values with the plant’s total numbers of flowers, fruits, leaves, and total new branch length, the total flower, fruit, and leaf biomass and new branch biomass of the plant were obtained.

The floral biomass and fruit biomass together constitute the sexual reproduction biomass, while the biomass of new branches and leaves together constitute the vegetative biomass. In this study, sexual reproduction biomass was used as the indicator for sexual reproduction, the number of ramets as the indicator for clonal reproduction, and vegetative biomass as the indicator for vegetative growth.

### 4.3. Abiotic Factors

#### 4.3.1. Topography

Topography variables include elevation, slope, aspect, and convexity. Elevation data for the plot were recorded using a GPS at the four corners of the plot, and the average value was then calculated. Slope was determined by calculating the average angular deviation between each angle of the four triangular planes formed by connecting three of the four corners and the horizontal plane, which provides the slope value for the plot. Aspect refers to the direction that the slope faces, with the direction due south defined as 0°, and due west, due north, and due east defined as 90°, 180°, and 270°, respectively, in a clockwise direction. Since aspect is a circular variable, we standardized it using a method [76] to reflect the northerly (cosA) and easterly (sinA) orientations of the slope (ranging from −1 to +1). These two variables increase with more northerly and easterly aspects, respectively. Convexity is defined as the difference between the elevation of the center of the plot and the average elevation of all adjacent plots.

#### 4.3.2. Soil Nutrients

In the four directions (east, south, west, and north) of the selected *Acer barbinerve* genets, avoiding plant roots and rocks, 100 g of soil from the 0–10 cm soil layer was collected using an iron shovel from each of the four directions. The soil from all four directions was placed in cloth bags and mixed into a single composite soil sample. The sample was air-dried and then passed through 2 mm and 0.15 mm sieves. The powder was compressed into thin disks (pressure: 6 t) using a benchtop powder pelletizer, placed in plastic-sealed bags, and labeled. Finally, the carbon, nitrogen, and phosphorus content of the soil (in units of mg·kg^−1^) was measured using the J200 LA-LIBS Laser Spectroscopic Element Analyzer (Applied Spectra, Fremont, CA, USA). To avoid interference between male and female plants, samples from males and females were measured separately.

### 4.4. Biotic Factors

We used interspecific competition intensity (CI) to represent biotic factors, as it reflects, to some extent, the species richness around the genets and the size of surrounding genets, which can effectively show the influence of surrounding species on the research subject. In this study, the Hegyi index [77] is used to quantify the interspecific competition intensity. The diameter at breast height (DBH) of *Acer barbinerve* genets is used to represent tree size, as shown in the following formula:

$$CI=\sum_{j=1}^{m}\frac{D_{j}}{D_{i}}\times \frac{1}{L_{ij}}$$

where *D_i_* is the DBH of *Acer barbinerve* genets, *D_j_* is the DBH of a competitor tree, *L_ij_* is the distance between the competitor tree and the *Acer barbinerve* genets, *m* is the number of competitor trees. A larger CI value indicates greater competition pressure on *Acer barbinerve* genets. Based on the coordinates of the trees in the plot, the distance between the competitor trees and the *Acer barbinerve* genets can be calculated. We use the fixed circular plot method to calculate the competition intensity of genets. According to previous studies, the competition radius for both male and female *Acer barbinerve* genets is 4 m [28]. All other species within this radius are considered competitor trees.

### 4.5. Data Analysis

We first performed descriptive statistical analyses on the biotic and abiotic factors within the study area, and employed ANOVA to examine significant differences in these factors between sexes (Table S1). To assess whether significant differences existed in the overall environmental conditions between males and females, principal component analysis (PCA) was conducted. Spearman’s correlation analysis was implemented to evaluate the nonparametric correlation strength between pairwise variables (Figure 2). Prior to analytical procedures, all predictor and response variables were standardized using Z-scores. All model predictors had a variance inflation factor (VIF) < 4 (Table S2), indicating that multicollinearity between predictor variables was not a concern. Given the unequal sample sizes between sexes that might influence statistical outcomes, we performed multiple regression analyses incorporating interaction terms between sex and topographic features, soil nutrients, and interspecific competition to evaluate the environmental impacts on biological individuals. For multivariate model construction, topographic variables were subjected to PCA, with the first two principal components (topo_PC1 and topo_PC2) (cumulatively explaining 67.4% of variance) being retained (Figure S1). Similarly, soil variables underwent PCA, where the first principal component (soil_PC1) (explaining 55.5% of variance) was selected (Figure S1). This dimensionality reduction preserved essential information while enhancing regression stability. To further investigate the differential effects of environmental factors (including topo_PC1, topo_PC1, soil_PC1, and interspecific competition) on sexual reproduction, clonal reproduction, and vegetative growth, we constructed grouped partial least squares path modeling (PLS-PM) using the “plspm” package. In this framework, sex served as the grouping variable, enabling path analysis to discern sex-specific responses in growth and reproductive strategies under environmental influences. All statistical analyses were conducted in R 4.3.3.

## 5. Conclusions

Both abiotic and biotic factors significantly influence sexual reproduction, clonal reproduction, and vegetative growth in *Acer barbinerve*, with distinct responses between sexes. Environmental variables, particularly topography and soil nutrients, interact differently with males and females, affecting their reproductive strategies and growth patterns. While female sexual reproduction is more sensitive to environmental factors, interspecific competition negatively impacts both male and female clonal reproduction. These findings highlight the complex interplay between abiotic and biotic factors in shaping the growth and reproductive strategies of dioecious plants in diverse environments.

## Data Availability

Data will be made available on reasonable request to the corresponding author.

## Supplementary Materials

The following supporting information can be downloaded at: https://www.mdpi.com/article/10.3390/plants14040596/s1, Table S1: Basic Information of the selected *Acer barbinerve* genets. Table S2: Effects of environmental variables on sexual reproduction, clonal reproduction, and vegetative growth. Figure S1: The biplot resulting from the principal component analysis for topography and soil nutrients.
