# Supplementary material for: Sex-Specific Responses of Sexual Reproduction, Clonal Reproduction, and Vegetative Growth to Environmental (Biotic and Abiotic) Factors in the Clonal Dioecious Plant Acer barbinerve
Source: Plants (Basel). 2025 Feb 15;14(4):596. doi: 10.3390/plants14040596 (PMC11860127; doi:10.3390/plants14040596)
Supplement: Supplementary file 1 [file plants-14-00596-s001.zip › Table S1.pdf]

Table S1: Basic Information of the selected*Acer barbinerve* genets.

Variables	Females			Males		
	Mean	Range	Standard deviation	Mean	Range	Standard deviation
C content of soil (mg/kg)	271.37 a	269.31~272.54	0.83	270.84 b	269.55~272.6	0.73
N content of soil (mg/kg)	357.75 a	332.21~365.75	7.31	355.40 a	343.57~362.86	5.87
P content of soil (mg/kg)	1451.75 a	1408.25~1491.78	23.10	1457.71 a	1413.39~1507.76	26.35
Elevation (m)	464.73 a	435.47~501.96	23.61	448.62 b	435.47~473.69	12.22
Slope (°)	10.78 a	2.98~25.27	6.23	8.72 a	1.36~20.87	6.17
Aspect (°)	238.60 b	160.24~286.29	44.93	230.35 b	168.72~267.49	30.67
Convexity degree	-0.11 a	-0.66~1.54	0.55	-0.16 a	-0.97~1.15	0.52
Sexual biomass (g)	45.52 a	1.89~127.02	35.70	2.61 b	0.61~6.78	1.59
Number of ramets	5.65 a	2~14	3.03	5.87 a	2~10	2.19
Vegetative biomass (g)	236.37 a	41.91~757.38	179.76	184.04 a	44.17~713.47	157.20
Interspecific competition intensity	12.38 a	1.88~41.55	11.75	8.17 a	0.16~26.87	7.22

For the same indicator, different lowercase letters indicate significant differences ($p < 0.05$).
