# Supplementary material for: Sex-Specific Responses of Sexual Reproduction, Clonal Reproduction, and Vegetative Growth to Environmental (Biotic and Abiotic) Factors in the Clonal Dioecious Plant Acer barbinerve
Source: Plants (Basel). 2025 Feb 15;14(4):596. doi: 10.3390/plants14040596 (PMC11860127; doi:10.3390/plants14040596)
Supplement: Supplementary file 1 [file plants-14-00596-s001.zip › Table S2.pdf]

Table S2: Effects of environmental variables on sexual reproduction, clonal reproduction and vegetative growth. We indicate the predictor estimate (Est.) and the variance inflation factor (VIF). gender, based on males; topo_PC1, the first axis of the five topography variables; topo_PC2, the second axis of the five topography variables; soil_PC1, the first axis of the four soil nutrients variables; CI, interspecific competition; gender:topo_PC1, the interaction between gender and the first axis of the five topography variables; gender:topo_PC2, the interaction between gender and the second axis of the five topography variables; gender:soil_PC1, the interaction between gender and the first axis of the four soil nutrients variables; gender:CI, the interaction between gender and interspecific competition.

	**sexual reproduction**		**clonal reproduction**		**vegetative growth**	
predictors	Est.	VIF	Est.	VIF	Est.	VIF
gender	-1.30	1.18	0.07	1.18	-0.49	1.18
topo_PC1	0.28	2.85	0.28	2.85	0.12	2.85
topo_PC2	-0.01	3.59	0.19	3.59	-0.19	3.59
soil_PC1	0.26	2.34	0.13	2.34	0.06	2.34
CI	-0.24	2.74	-0.43	2.74	-0.29	2.74
gender:topo_PC1	-0.29	2.68	-0.23	2.68	-0.28	2.68
gender:topo_PC2	0.004	3.27	-0.31	3.27	0.03	3.27
gender:soil_PC1	-0.25	1.87	-0.20	1.87	-0.11	1.87
gender:CI	0.22	2.78	0.002	2.78	-0.12	2.78
