# Supplementary figures and images for: Sex-Specific Responses of Sexual Reproduction, Clonal Reproduction, and Vegetative Growth to Environmental (Biotic and Abiotic) Factors in the Clonal Dioecious Plant Acer barbinerve
Source: Plants (Basel). 2025 Feb 15;14(4):596. doi: 10.3390/plants14040596 (PMC11860127; doi:10.3390/plants14040596)

(a) PCA of topography

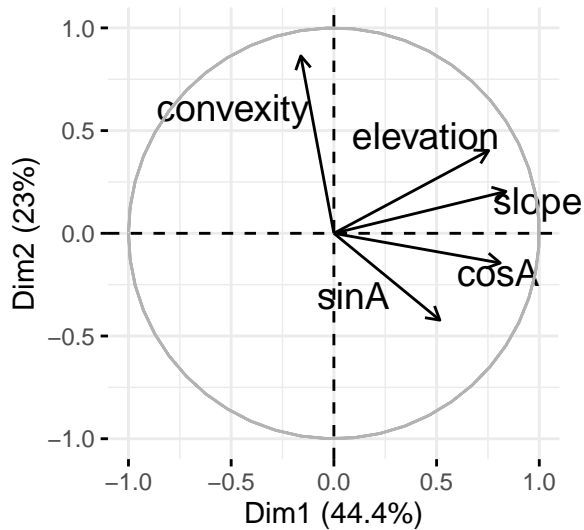

(b) PCA of soil nutrients

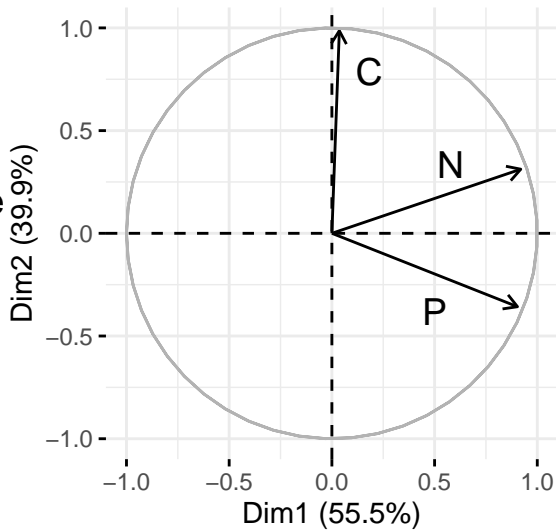

Supplement: Supplementary file 1 [file plants-14-00596-s001.zip › Figure S1.pdf]
